# Embodied Computational Evolution: A Model for Investigating Randomness and the Evolution of Morphological Complexity

**DOI:** 10.1093/iob/obae032

**Published:** 2024-08-21

**Authors:** E Aaron, J H Long

**Affiliations:** Department of Computer Science, Colby College, Waterville, ME 04901, USA; Interdisciplinary Robotics Research Laboratory, Vassar College, Poughkeepsie, NY 12604, USA; Department of Cognitive Science, Vassar College, Poughkeepsie, NY 12604, USA; Interdisciplinary Robotics Research Laboratory, Vassar College, Poughkeepsie, NY 12604, USA; Department of Cognitive Science, Vassar College, Poughkeepsie, NY 12604, USA; Department of Biology, Vassar College, Poughkeepsie, NY 12604, USA

## Abstract

For an integrated understanding of how evolutionary dynamics operate in parallel on multiple levels, computational models can enable investigations that would be otherwise infeasible or impossible. We present one modeling framework, *Embodied Computational Evolution* (*ECE*), and employ it to investigate how two types of randomness—genetic and developmental—drive the evolution of morphological complexity. With these two types of randomness implemented as germline mutation and transcription error, with rates varied in an $11\times 11$ factorial experimental design, we tested two related hypotheses: (***H_1_***) Randomness in the gene transcription process alters the direct impact of selection on populations; and (***H_2_***) Selection on locomotor performance targets morphological complexity. The experiment consisted of 121 conditions; in each condition, nine starting phenotypic populations developed from different randomly generated genomic populations of 60 individuals. Each of the resulting 1089 phenotypic populations evolved over 100 generations, with the autonomous, self-propelled individuals under directional selection for enhanced locomotor performance. As encoded by their genome, individuals had heritable morphological traits, including the numbers of segments, sensors, neurons, and connections between sensors and motorized joints that they activated. An individual’s morphological complexity was measured by three different metrics derived from counts of the body parts. In support of ***H_1_***, variations in the rate of randomness in the gene transcription process varied the dynamics of selection. In support of ***H_2_***, the morphological complexity of populations evolved adaptively.

## Introduction

The evolution of populations is driven by three general types of causes: randomness, history, and adaptation (Rebolleda-Gómez and Travisano 2019; Travisano et al. 1995). Randomness is part of nearly every process used by living organisms, including genetic mutation, recombination, gene-to-phenotype mapping, physiology, behavior, and even natural selection itself (Wagner 2012). In the context of adaptation, randomness is a part of nearly every process that impacts the evolutionary trajectory of living organisms (Lynch 2010). In spite of the fact that mutation’s role as a source of genetic variation is well established, less clear is how it interacts with selection “in driving evolutionary divergence” (Svensson and Berger 2019). In the context of development, randomness also intervenes, operating as *stochastic developmental variation* (*SDV*, Vogt 2015), a mechanism separate from the adaptive environmental responses of developmental plasticity (West-Eberhard 2003). While long thought to be an unavoidable drag on selection efficiency, SDV not only builds phenotypic variation from a common genotype but may also do so via processes that are themselves heritable (Vogt 2015). While a model organism—the marbled crayfish, *Procambarus virginalis*, an obligate asexual species—has emerged for studying the genetic and epigenetic bases of developmental plasticity, whether SDV and selection interact causally to drive evolution remains unknown in animal studies (Vogt 2023).

Given the challenges using organisms to study how SDV and mutation may interact with selection—and, by extension, with each other—we have created the *Embodied Computational Evolution* (*ECE*) modeling framework (Hawthorne-Madell 2021). Evolving simulated asexual animals—*biorobots*, in the sense of (Webb 2001)—under a variety of experimental conditions, we generated a database of more than 6 million individuals. Here, we undertake a new analysis of those experimental results. Specifically, we aim to (1) test hypotheses about the relative roles and interaction of mutation, SDV, and selection on the evolution of morphology and complexity, and, in so doing, (2) demonstrate the explanatory power of ECE as a modeling framework for biologists.

When mutation and development influence body morphology, these two types of randomness have the potential to directly impact behavior. Locomotion, in particular, is a foundational behavior in animals that depends on the body interacting with the environment to transfer momentum. In addition, locomotion in multicellular animals requires that various components of the body—sensors, nervous system, muscles, segments, and limbs—interact and coordinate movement. Thus, it’s not surprising that body morphology and its variable complexity have been the focus of a recent surge of research as investigators reconstruct ancestral body forms (Schwaha et al. 2020), seek and establish direct links between fitness and body morphology (Hager and Hoekstra 2021; Le Roy et al. 2019; Ord 2020), and build an understanding of morphospace and adaptive landscapes (Aaron et al. 2022; Price et al. 2019).

Morphological complexity itself has been hypothesized to increase via the historical “first law” effect of lineages accumulating random variations (McShea et al. 2019). In critiquing how biologists study the evolution of complexity, McShea (2021) highlights the need for an understanding of complexity that allows us to actually measure it in organisms and, then, to measure changes in complexity over evolutionary time. We take on both priorities in this paper. We measure morphological complexity in three simple ways: (1) external *mechanical complexity*, the number of body segments; (2) internal *sensorimotor complexity*, the number of sensors, neurons, and components connecting them; and (3) *total complexity*, the sum of the mechanical and sensorimotor complexities. These are “horizontal” complexities (McShea 2021), existing at one structural level in the organism. We further address McShea’s highlighted priorities by running experiments, selecting individuals in each biorobotic population for enhanced locomotion, and, in response, measuring how morphological complexity evolves.

When experiments on living organisms reach their practical limits, computational and formal models can extend the reach of biologists to investigate complex systems. Genetics and genetic interactions, for example, have been examined in populations of individuals using digital organisms (Lenski 1999). Stemming from this seminal work, a family of *in silico* experiments on digital organisms addressed questions related not only to genetics, but also to historical contingency, the evolution of complexity, and the mechanisms of phenotypic plasticity (for review, see Fortuna et al. 2022). Within population genetics, empirical models include statistical approaches, with the interaction between genetics and development quantified at the level of the evolution of a population’s genetic variance–covariance matrix, to simultaneously investigate multiple traits under selection (Arnold 1992; Cheverud 1984).

For population genetics, the study of mutations in an evolutionary context is central. In their seminal review, Loewe and Hill (2010) note:

A major theoretical goal in the study of the population genetics of mutations is to understand how mutations change populations in the long term. To this end, we have to consider many features of evolution and extant populations at both the phenotypic and molecular level, and ask how these can be explained in terms of rates and kinds of mutations and how they are affected by the forces that influence their fates.

Achieving this goal requires an integrative approach—spanning molecules, organisms, and populations—that simultaneously incorporates the complexity created by the parallel operation of different evolutionary forces (Loewe and Hill 2010). Evolutionary consequences of mutation can be understood by examining the distribution of fitness effects (Charlesworth 1996; Eyre-Walker and Keightley 2007). Because mutations occur in different ways, genes interact, and a single gene can have many different effects, the mutational effects on fitness vary from beneficial to deleterious. In the case of coloration in animals, mutations have wide-ranging effects on the adaptive value of ecologically relevant behaviors such as social signaling among conspecifics and predator avoidance through visual camouflage, aposematism, or mimicry (for review, see Orteu and Jiggins 2020).

To examine a population’s behavior from the explanatory level of its constituent individuals, ecologists use *agent-based models* (*ABMs*) (Avin et al. 2021). Within the movement ecology framework (Nathan et al. 2008), ABMs explicitly model individual agents and their cognitive states, decision-making, navigational capacities, and motion capacities (for review, see Tang and Bennett 2010). However, individuals in these models lack realistic physics-based self-propulsion, an essential element of autonomous behavior.

**Fig. 1:**
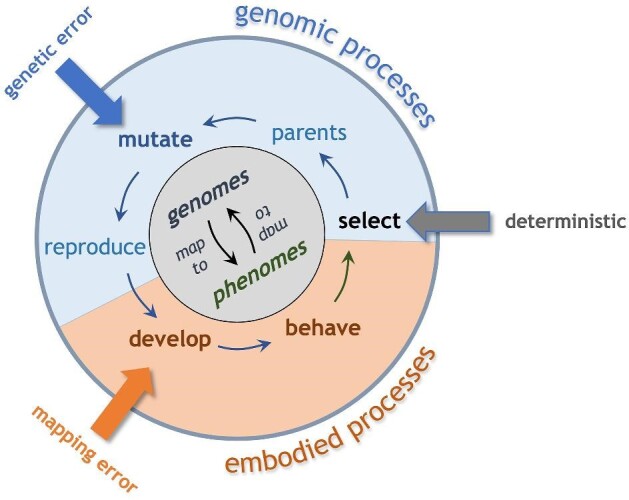
ECE is an agent-based modeling framework that incorporates the entire life cycle of each individual in a population of those individuals. Embodied processes include the development of the individual and its behavior in an environment. Behavioral differences among the individuals in a population are used deterministically to select the parents in the population for reproduction. Genomic processes may include the mutation of the genome that is used in reproduction. The genome is expressed in the developmental process. Random errors can, in principle, be introduced at any place in this life-cycle.

To augment conventional ABMs, ECE offers an integrative framework that allows us to conduct *in silico* experiments on populations of behaviorally autonomous, self-propelled organisms with bio-realistic morphology and reconfigurable bodies (Fig. 1). Individuals have bodies with physically realistic properties, with morphology and movement subject to the laws of physics; this is “embodiment” in the sense of ECE. In this paper, we commonly call these individuals “biorobots” because they are embodied models built to test biological hypotheses, in the manner of Webb’s biorobotic paradigm (Webb 2001). In ECE models, populations are composed of individuals with the following features: genomes with genetic interactions; development with fixed and/or variable rules; physically embodied morphology with sensorimotor circuits that drive self-propulsive body reconfigurations; and behavior that is autonomously generated by the organism interacting with its physical environment.

In our first analysis of the experimental results of this ECE model (Hawthorne-Madell 2021), we tested and found support for the *masquerading genome hypothesis* (*MGH*): The presence of SDV, realized as randomness in gene transcription, can shield genes from the direct impact of selection, thus increasing genetic variance in evolving populations. In this paper, we refine the MGH and take on McShea’s challenge of measuring the evolution of complexity in two hypotheses (and corresponding predictions):

- ***H_1_***: Randomness in the gene transcription process alters the direct impact of selection on populations.
- ***P_1_***: Variations in the rate of randomness in the gene transcription process will vary the dynamics of selection.
- ***H_2_***: Selection on locomotor performance targets morphological complexity.
- ***P_2_***: The morphological complexity of populations will evolve adaptively, as detected by the presence of non-zero selection gradients.

These hypotheses would be refuted if (1) the selection gradients on traits are invariant with respect to the rate of transcription error and (2) morphological complexity is not associated with non-zero selection gradients.

Encompassing these hypotheses, we used our ECE model to broadly investigate how the two different types of randomness—germline mutation and transcription error—influence the evolution of morphological complexity. We ran selection experiments by measuring the locomotor performance of individuals in evolving populations: Following development, each individual propelled itself on a flat surface, and the linear distance it traveled over a fixed period was used as a proxy for individual absolute fitness. The experimental design was fully factorial $11 \times 11$, with 11 levels of rate of transcription error and 11 levels of germline mutation rate for a total of 121 different conditions. In each condition, nine replicate populations of 60 individuals were evolved over 100 generations. This experiment generated a database of 6,534,000 individuals, with information logged about each individual’s genome, morphology, and locomotor performance. The results support both hypotheses and reveal that the evolutionary dynamics of morphological complexity can be altered by changes in and interactions of the rate of transcription error, the rate of germline mutation, and generational time.

## Our ECE model

**Fig. 2:**
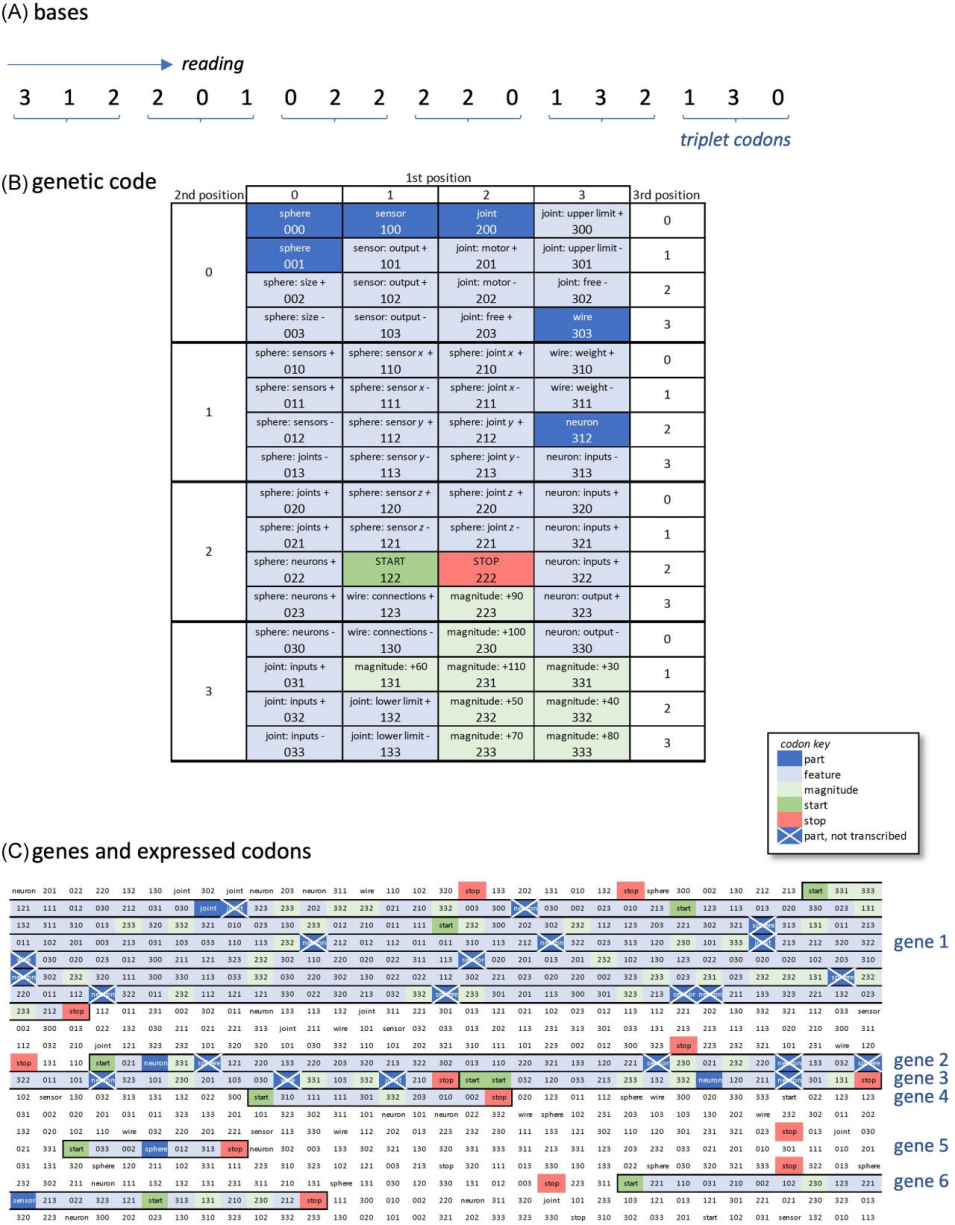
Genome for ECE individuals. **(A)** The simple genome is a continuous, single strand of 18k quaternary bases. During transcription, the quaternary bases, coded as integers 0–3, are read as triplet codons. **(B)** The genetic code. **(C)** Genes are defined from a start codon to a stop codon. In this example of a portion of a genome, six genes occur and five (genes 1–3, 5, 6) express a part, while one (gene 4) expresses only feature and magnitude codons. Part codons in a gene but not expressed are demarcated (crossed out).

**Fig. 3:**
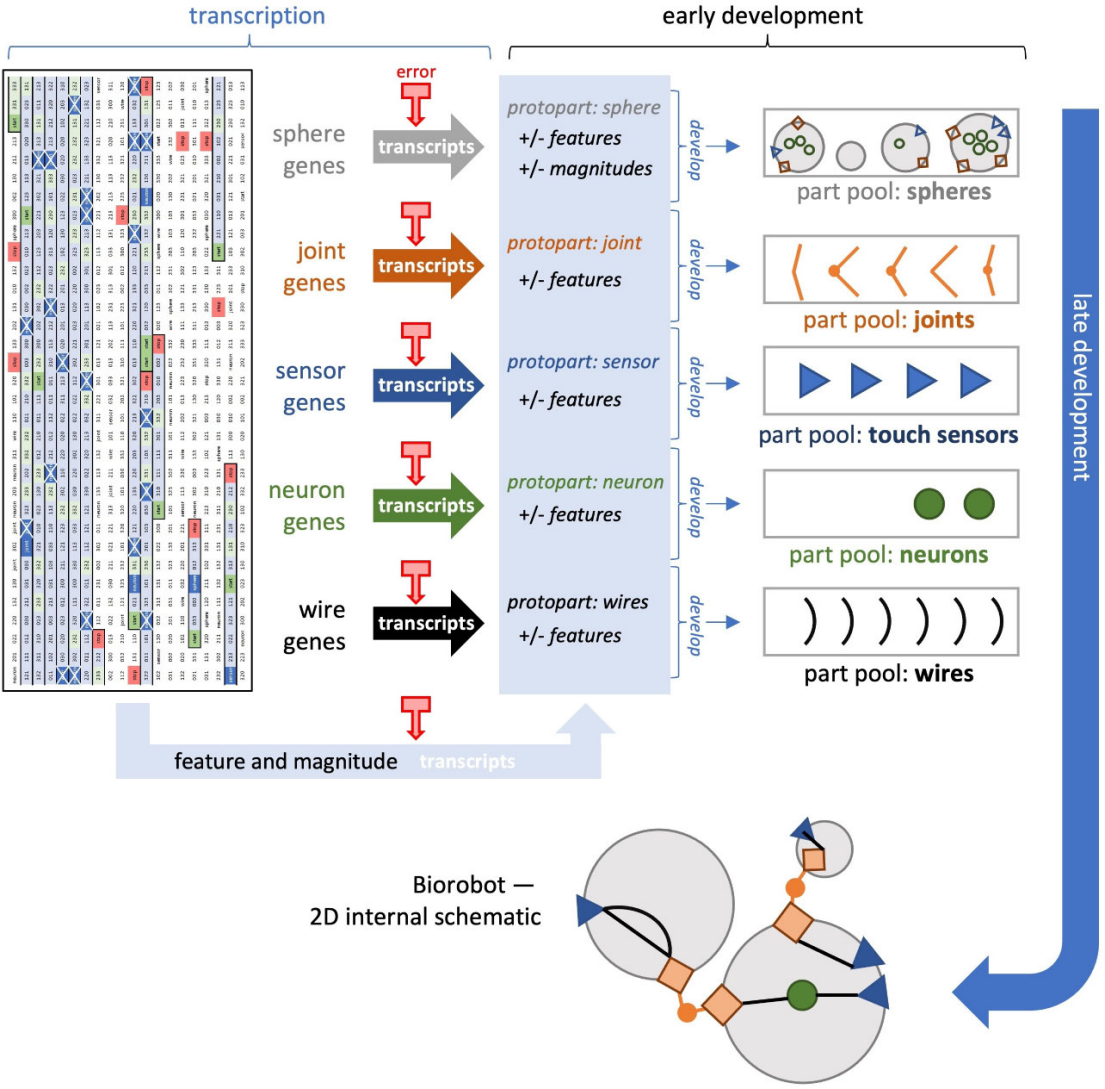
Development maps the genome to phenotypic parts, allowing for transcription errors in the process. Transcription is the first stage, with each gene producing a single part and multiple regulatory elements (feature and magnitude codons). Transcription errors (arrows with box end) may occur at this stage, and when present, they may convert one type of transcript into another, thus altering the mapping of genotype to phenotype. Parts and regulatory elements work together in early development to create the finished parts, which are then placed into part pools. From the parts, the whole biorobot is constructed as spheres (segments) connected by joints and populated with touch sensors, neurons, and wires.

**Fig. 4:**
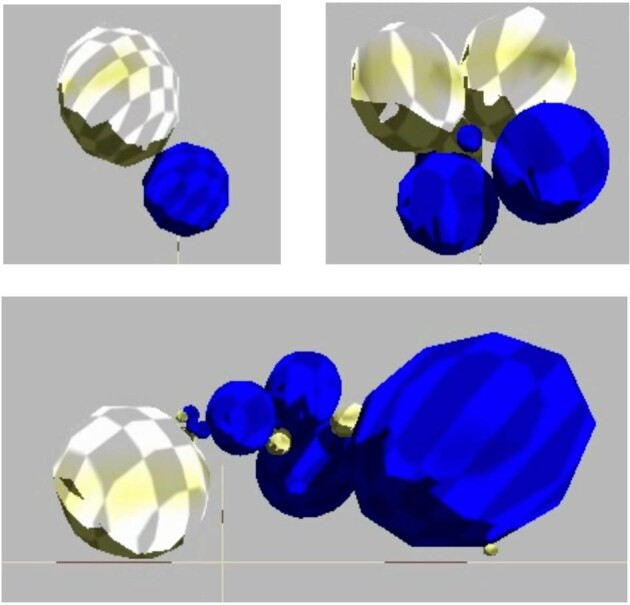
Behaviorally autonomous biorobots. The finished products of the genome-to-phenome mapping process are as varied as their genetic diversity and mapping errors. Here, we see simple forms as well as more complicated forms with many segments. They locomote on a flat, featureless surface, and obey the laws of the simulated physics. We use their locomotor performance as a proxy for their relative individual fitness in the selection algorithm that picks, each generation, the best 30 out of the population of 60 to reproduce.

While some details of our ECE model have been published elsewhere (Aaron et al. 2022; Hawthorne-Madell 2021), here, we review how the ECE model and our biorobots reflect underlying biological inspirations. As foundations for morphology, we chose body forms that are built from spherical segments. Segmented body plans may be ancestral for bilateral organisms (Couso 2009; Davis and Patel 1999). Thus, we modeled a body architecture, developmental process, and genomic structure that allowed for the evolution of the number, size, and arrangement of body segments (Hawthorne-Madell 2021). In addition to their segmented morphology, biorobots have biological foundations that include genomes with 18,000 quaternary bases, a genetic code (Fig. 2), and simple, explicitly modeled gene expression; development starts with transcription, includes processes for assembly, and produces the finished adult biorobot (Fig. 3). These processes, when coupled with germline mutation and errors of transcription, produce a variety of body morphologies that crawl, wriggle, and jump to locomote on a flat, empty, and terrestrial environment (Fig. 4).

Our ECE model does not itself explicitly govern the simulated physics of motion of our biorobots’ bodies; instead, that is fully controlled by an external physics engine, which determines the behavior of the individuals in the environment. In our view, the specific physics engine chosen to be employed for a given set of experiments is an implementation detail, rather than inherent to the ECE model. We chose to use a well established, external physics engine (see below) to provide a measure of validity to our biorobots’ motion, enabling us to assume that biomechanical movement and locomotion behavior are physically realistic at the level of Newtonian mechanics.

### Our biorobots: genetics

Each biorobot’s genome is a continuous, single strand of 18,000 bases, the size of some RNA viruses (Lynch 2010); a single strand of bases is used because of the relative simplicity it affords, compared to double-stranded structures. As with the biological genome, there are four possible bases. Here, we refer to them abstractly as the *quaternary* digits 0, 1, 2, 3 rather than as letters suggesting any particular nucleotide bases (e.g., A, C, G, T). Bases are part of genes when they occur between start and stop codons (Fig. 2). Codons are triplets according to the rules of the genetic code. There are a total of 64 different codons; note that as in the biological genetic system, there is some redundancy in coding, so that, e.g., 000 and 001 both code for a spherical segment.

When expressed as transcripts, codons dictate the parts that comprise the biorobots’ morphologies and alter development of those parts by adjusting their features and the magnitudes of their growth. Each gene codes for at most one of the five kinds of parts from which the biorobots are built: Segments (spheres), joints, sensors (i.e., touch sensors), neurons, and wires. A genome can contain multiple genes (Fig. 2C). Multiple triplet codons for body parts (called *part codons*) can occur between the start and stop codons of a gene; in those cases, only the first of the part codons is expressed in development. Although in our experiments, the length of each genome is fixed at 18,000 bases for every individual in every generation, mutation could potentially alter any codon during reproduction, so the length and number of genes within a genome can evolve.

### Our biorobots: development and morphology

The G–P map of our biorobots can be considered to be a three-stage developmental process (Fig. 3):

- *transcription*, in which genes are transcribed into precursors to parts, which we call *protoparts*, and regulatory elements;
- *early development*, in which protoparts develop and are expressed as parts, which can be components of a fully developed biorobot; and
- *late development*, in which the biorobot is assembled from the finished parts.

The transcription stage is, in the absence of random errors, the straightforward mapping of each gene in a genome to the protopart corresponding to the gene’s expressed part codon—e.g., if the part codon to be expressed is a sphere codon, transcription creates a sphere protopart. Random errors, however, could result in transcription creating a protopart not corresponding to the expressed part codon of the gene—e.g., if the part codon to be expressed is a sphere codon, transcription errors could result in a joint, sensor, neuron, or wire protopart. As a methodological note, in this paper, these transcription errors are the only occurrence of randomness in the developmental process (i.e., as distinct from randomness in genomic mutation); in principle, randomness could have been added to other stages of development.

The early development stage then enables the transcribed regulatory elements, as coded by feature and magnitude codons, to be represented in the development of each protopart into a part, finalized as a potential component in the biorobot assembled in late development.

Finally, the late development stage results in the construction of our biorobots’ bodies, which are branched and segmented, composed of spherical segments connected by joints. For simplicity and focus, our ECE model abstracted away from details of how segmentation might arise developmentally from lower-level processes (e.g., molecular mechanisms), instead explicitly encoding in our development algorithm that only branched and segmented body forms could result. As determined by the genome and the G–P map, the size, number, and orientation of segments can vary from individual to individual, as can features of the parts that comprise a body. Each segment may have a variable number of mounts for joints, touch sensors, or neurons. Wires are analogous to nerves in the morphology of our biorobots, connecting sensors to motors or neurons, or connecting neurons to each other. A sensor may initialize movement if its touch signal is transmitted via a sensorimotor circuit to a motorized joint, which can occur in two ways: A wire connects the sensor directly to the motorized joint; or a wire connects the sensor to a neuron (group) that connects to the motorized joint.

In late development, assembly begins with the mechanical morphology, connecting segments with joints to form one or more branches, conditional upon available resources of segments, joints, and joint mounts on segments. Segments are added in series, elongating the initial branch when possible, with each newly added segment becoming the active point for the next step. If the active segment lacks an available joint mount in the presence of a new segment and new joint, the process switches from elongation to branching; this is the only context in which a new branch may be formed. Proceeding from the original segment in order of connection, the algorithm looks for an available joint mount. The first available mount receives the new joint and segment, creating a new branch. This new branch is then elongated until branching is required. Elongation and branching swap in that order until one of these conditions terminates construction of the mechanical morphology: (1) no unattached segments remain; (2) no joints remain; or (3) no open joint mounts remain.

Following mechanical morphology assembly, the sensorimotor morphology is assembled by a similar process that is once again conditioned on available resources. Neurons and sensors are first added to open mounts until parts or mounts are depleted; wires are then added until parts or open connectors are depleted. In sum, the sensorimotor morphology can be thought of as “internal,” encompassing the sensors, wires, and neurons that pass information from activated sensors to motorized hinges. In a complementary fashion, the mechanical morphology can be thought of as “external,” encompassing the segments that transfer momentum to the ground to produce locomotion.

## Experiments

In the experiments, we varied values of germline mutation rate $\mu$ and transcription error rate $\tau$ from 0 to 0.0050, in increments of 0.0005, representing the probability per generation of a change in an individual’s quaternary genomic base or a transcript codon base, respectively. At the highest rate, this yields, on average, 90 genomic and 90 transcriptomic base changes per individual, keeping in mind that all individuals bear an 18 kB genome. We implemented germline mutation as a random process operating as point mutations of the genome when duplicating the genome during reproduction. We implemented transcription error as a random process operating as a point mutation of the codon during the first step of phenotypic development (Fig. 3). In this manner, mutation and transcription error are independent processes, one genetic and one developmental, and can be manipulated separately in experiments. The outcomes of these two processes interact at the level of the developed phenotypes, the biorobots, and their populations; for example, when $\tau$ increases and $\mu$ is held constant, then we expect a decrease in the traits’ narrow-sense heritability, the ratio of additive genetic variance to total phenotypic variance.

The highest rate of $\mu$ falls in the range that was measured in RNA viruses, $10^{-3}$–$10^{-6}$ (Lynch 2010). While the size of the genome was fixed throughout the experiments, the number, size, and composition of genes were evolvable features of the genome. The ranges and intervals of variation for the two types of randomness were identical, allowing a direct comparison of their evolutionary effects. With 11 magnitudes of $\mu$ and 11 magnitudes of $\tau$, all pairwise combinations were run to yield 121 different randomness conditions.

Because the units of evolution are by definition populations, we organized the experiments around them. We created 9 different founding genome populations of 60 individual genomes each, where each genetic base was randomly selected from a uniform distribution, with replacement, of the four bases. In generation 0, before selection and reproduction, the distribution of Hamming distances of the nine populations were statistically indistinguishable from their mean in a uniform distribution ($p> 0.05$, $\chi ^2$ test); the phenotypic variances of the starting biorobotic populations were statistically indistinguishable ($p> 0.05$ in a one-way analysis of variance) at $\mu$ and $\tau =0$ for all four response variables: absolute fitness, total complexity, mechanical complexity, and sensorimotor complexity. Each of the nine founding genome populations represents a “subject” in a statistical sense: individuals developed from those genomes and within a biorobot population will covary more with each other over generational time than they will with individuals in other biorobot populations. This subject effect is addressed in linear mixed-effects models (LMMs) by treating the subject effect as a random variable and distinguishing between within-subjects and between-subject independent variables.

Each of the nine founding genome populations was used to create 121 phenotypic populations of 60 biorobots, with each of those populations assigned to one of the 121 experimental conditions of randomness. A given founding genome population’s 121 biorobot populations were, in generation 0, genetically identical but phenotypically different. Those initial phenotypic differences arose from development (see previous section): To produce a biorobot, each genome was mapped to its phenotype, a process that includes (except when $\tau =0$) random transcription errors. In generation 0, at the 10 levels where $\tau > 0$, the transcriptional errors present during development create, by design, different phenotypic starting points for each biorobotic population for a given founding genome population.

Each of the 1089 starting biorobot populations (9 founding genome populations × 121 experimental conditions) was evolved over 100 generations. With population size limited to 60 individuals, this design yielded a possible total of 6,534,000 individual biorobots. The actual number of individuals evolved was 6,383,554. A total of 149,889 individual biorobots were removed when they were assigned an absolute fitness of 0 because they were unable to move or to be assembled. For example, immobility happens when (1) spheres cannot be created (one or less sphere genes), (2) spheres cannot be connected (insufficient joint genes), (3) sensors are absent (insufficient sensor genes), or (4) neural networks cannot connect a sensor to a motorized joint (insufficient wire genes or insufficient connections). An additional 17 individuals were assigned a value of *NA*, rather than 0, for their absolute fitness. Finally, in what appears to be a undetected date recording error during file writing, all 540 individuals from generation 91–99 in population 2, evolving with a $\mu$ of 40 and a $\tau$ of 50, lack data.

Each of the 6,383,554 viable biorobots was evaluated in a behavior task. Using a physics engine (Bullet v. 2.82, pybullet.org) for realistic simulated motion, each individual was placed on a flat, hard, and featureless substrate. The initial interaction with the substrate activated touch sensors and generated ground-reaction forces, which, in turn, caused movement driven by motorized joints and inertia of the body. After 501 time steps, the behavioral performance was measured as the linear, two-dimensional distance from the start to the end of the movement.

This behavioral performance was used in two ways: (1) to approximate an individual’s absolute fitness, a property that may be compared among populations and across generations given certain strict assumptions (as stipulated by (Wilson 2004) and met by this model) and (2) to approximate an individual’s relative fitness, which is used in a given population and generation to create differential reproduction. Reproduction was asexual, a mode chosen because of its relative genetic simplicity compared to sexual reproduction (Aaron and Long 2021). For each population and generation of 60 biorobots, the 30 individuals with the highest performance values were selected for reproduction. Within that set, performance rank determined relative individual fitness:

- individuals ranked 1–3 (i.e., having one of the three highest performance values): four offspring each (for a total of 12 from these three parents);
- individuals ranked 4–9: three offspring each;
- individuals ranked 10–18: two offspring each;
- individuals ranked 19–30: one offspring each.

After reproducing 60 offspring genomes, parents died. Thus, this truncation selection and rank-assigned differential reproduction created a maximum population size of 60. During reproduction, offspring genomes were created by simple duplication of the single-stranded parental genome and subsequent random mutation of bases. As mentioned at the beginning of this section, the rate of mutation, $\mu$, varied as an experimental factor. All simulations were run on a System76 laptop, model Gazelle Professional, with eight Intel® CoreTM i7-4710MQ CPUs @ 2.50GHz within an Ubuntu 14.04.4 LTS x86 64 environment.

To investigate morphological complexity, we created three simple metrics:

- *mechanical complexity*: As a measure of complexity for an individual’s mechanical morphology, we used the *number of segments* in that individual. As a methodological note, we could instead have incorporated the number of joints *j* in that individual along with the number of segments *s*, but we chose the simpler measure because *s* and *j* are not independent: $j = s - 1$ in every individual.
- *sensorimotor complexity*: As a measure of an individual’s sensorimotor morphological complexity, we use the sum of three quantities: (the *number of sensors* in that individual) $+$ (the *number of neurons* in that individual) $+$ (the *number of wires* in that individual).
- *total complexity*: As a measure of an individual’s total morphological complexity, we use the sum of that individual’s mechanical complexity and sensorimotor complexity measures.

For visualization, trends over generational time and among variables of interest were determined with a moving local regression method, the geom_smooth function in the ggplot2 library of R, with formula $y \sim x$, method *loess*, and default settings *span*$= 0.75$, *degree*$= 2$. We regressed each response variable onto generation, pooling data across $\tau$ to examine the effects of $\mu$ and pooling data across $\mu$ to examine the effects of $\tau$. Each regression line was plotted with a 95% confidence interval. In some visualizations, we examined the effects of the population on evolutionary trajectory using the geom_path function.

For statistical modeling, we sought to investigate the three-way interactions among generation, $\mu$, and $\tau$. Using an LMM, $\mu$, $\tau$, and generation were within-subjects variables and population was the subject. Using the lmer function of the lme4 library in R, REML, each response variable was examined, testing the main effects and all interactions among the three independent variables.

## Results

### Absolute fitness

**Fig. 5:**
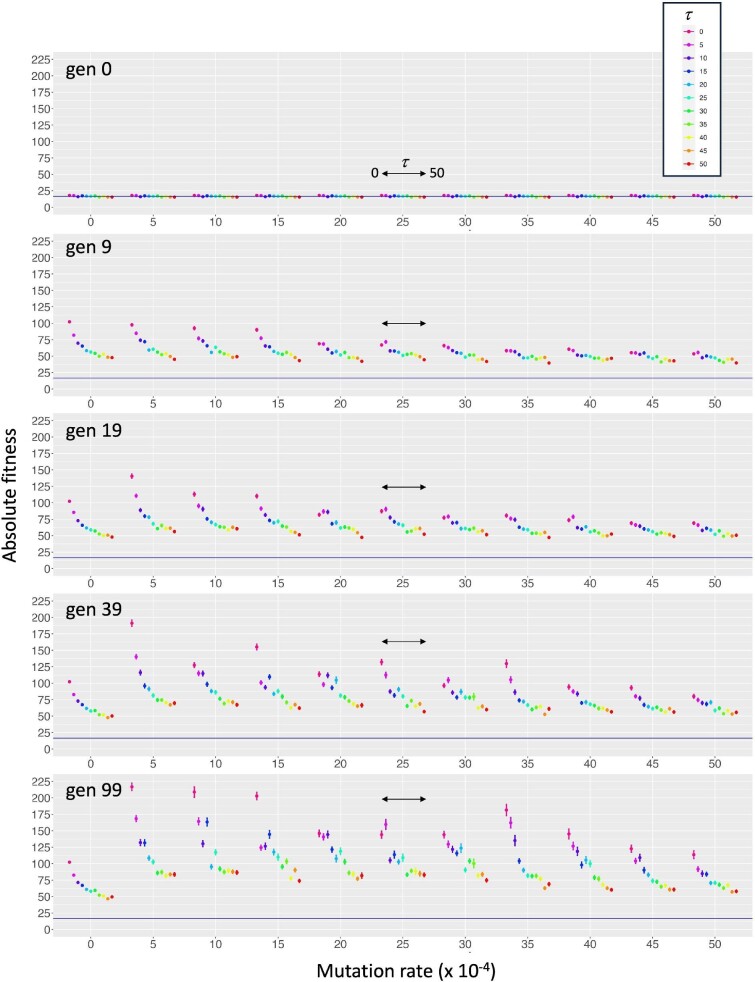
Mutation rate ($\mu$) and transcription error rate ($\tau$) interact, and do so over generational time, as shown by absolute fitness. Those three factors interact in a significant three-way interaction ($p < 0.00001$, linear mixed model; this effect occurs in all response variables, see Table 1). That interaction is shown here as the change in position, range, and shape of the $\tau$ curve shown at each value of $\mu$ at the five representative generations. The constant absolute fitness in generation 0, measured in randomly generated individuals in populations that have yet to be exposed to selection, provides a reference (blue horizontal line) for evolutionary changes in the subsequent representative generations. All 100 generations were analyzed in the statistical model; the five shown here were chosen to correspond with the start, rapid peak, rapid decline, and then relative stability of the complexity metrics over generational time (see Fig. 6). Means $\pm 1$ st. error ($n=540$ for each mean). Double-headed arrows represent the smaller scale of $\tau$ at each $\mu$; the scale for $\tau$ is the value depicted $\times 10^{-4}$.

Measured directly from locomotor performance, absolute fitness is expected to change under selection, and it does (Fig. 5). As selection acts from generation 1 to 99, all mean values (taken from all 60 individuals from 9 populations) of the 121 conditions (11 $\mu \times$ 11 $\tau$) rapidly exceed the fitness from generation 0.

However, the patterns are complicated with respect to generation, $\mu$, and $\tau$, which reflect a significant three-way interaction ($p < 0.00001$; see Table 1 for complete results). This interaction can be seen as the change in the position, range, and shape of the curve of absolute fitness with respect to $\tau$ at different values of $\mu$ at different generations. In generation 9, the lowest values of $\mu$, 0–15 ($\times {10}^{-4}$), show the greatest range of $\tau$ values and a curvilinear shape that distinguishes them from the lower-range and straight $\tau$ curves at higher values of $\mu$. By generation 39, that pattern has changed, with the largest ranges occurring at $\mu$ values of 5, 10, 15, and 35, and the curvilinear shape is apparent at all values of $\mu$ except 30 and 40. Except when $\mu$ is zero, variance of absolute fitness, as shown by the standard error of the mean, increases over generational time at every combination of $\mu$ and $\tau$. At any given value of $\mu$, the smallest variances are seen at the highest values of $\tau$. But at the highest values of $\mu$, 45 and 50, there is less variance at a given value of $\tau$ than at levels of $\mu$ from 10 to 40.

**Table 1:** Linear mixed effects models of genetic variance (as Hamming distance) and the four response variables, with fixed effects shown

	Estimate	Std. Error	df	*t* value	Pr($> \!\!|t|$)	
**Genetic variance**						
(Intercept)	5671e+06	2.024e+06	9.847e+03	2.802	0.00509	**
generation	$-$ 8.160e+04	2.769e+04	4.814e+02	$-$ 2.947	0.00337	**
$\mu$	1.183e+09	3.091e+07	1.078e+05	38.281	$< $ 2e$-$16	***
$\tau$	5.612e+08	3.092e+07	1.073e+05	18.151	$< $ 2e$-$16	***
generation $\times \mu$	$-$ 5.598e+06	5.391e+05	1.089e+05	$-$ 10.384	$< $ 2e$-$16	***
generation $\times \tau$	$-$ 7.410e+06	5.390e+05	1.089e+05	$-$ 13.747	$< $ 2e$-$16	***
$\mu \times \tau$	$-$ 1.027e+11	1.044e+10	1.089e+05	$-$ 9.837	$< $ 2e$-$16	***
generation $\times \mu \times \tau$	1.901e+09	1.823e+08	1.089e+05	10.430	$< $ 2e$-$16	***
	Estimate	Std. Error	df	*t* value	Pr($> \!\!|t|$)	
**Absolute fitness**						
(Intercept)	8.25E+01	2.59E+01	1.39E+02	3.187	0.00178	**
generation	8.42E-01	4.12E$-$02	6.21E$-$01	20.432	0.09613	
$\mu$	$-$ 7.59E-01	3.08E+01	5.14E$-$04	$-$ 0.025	0.99952	
$\tau$	$-$ 8.88E-01	3.05E+01	1.77E+00	$-$ 0.029	0.97970	
generation $\times \mu$	6.14E-04	1.12E$-$04	6.38E+06	5.483	4.18E $-$08	***
generation $\times \tau$	$-$ 1.11E-02	1.12E$-$04	6.38E+06	$-$ 99.260	$< $ 2.0E$-$16	***
$\mu \times \tau$	1.65E-02	2.17E$-$04	6.38E+06	75.977	$< $ 2.0E$-$16	***
generation $\times \mu \times \tau$	$-$ 4.13E-05	3.79E$-$06	6.38E+06	$-$ 10.904	$< $ 2.0E$-$16	***
	Estimate	Std. Error	df	*t* value	Pr($> \!\!|t|$)	
**Total complexity**						
(Intercept)	2.28E+01	2.31E+00	3.15E+02	9.871	$< $ 2.0E$-$16	***
generation	1.29E-02	4.94E-03	2.69E+00	2.615	0.08880	
$\mu$	$-$ 2.56E$-$02	2.87E+00	1.47E$-$03	$-$ 0.009	0.99970	
$\tau$	$-$ 4.61E$-$03	2.75E+00	2.56E$-$04	$-$ 0.002	1.00000	
generation $\times \mu$	$-$ 6.45E$-$04	9.94E$-$06	6.38E+06	$-$ 64.871	$< $ 2.0E$-$16	***
generation $\times \tau$	$-$ 4.60E$-$04	9.94E$-$06	6.38E+06	$-$ 46.300	$< $ 2.0E$-$16	***
$\mu \times \tau$	9.86E-04	1.93E$-$05	6.38E+06	51.059	$< $ 2.0E$-$16	***
generation $\times \mu \times \tau$	1.36E-05	3.37E$-$07	6.38E+06	40.341	$< $ 2.0E$-$16	***
	Estimate	Std. Error	df	*t* value	Pr($> \!\!|t|$)	
**Mechanical complexity**						
(Intercept)	5.59E+00	7.56E$-$01	2.70E+00	7.398	0.00729	**
generation	$-$ 8.45E$-$04	1.43E$-$03	3.89E+00	$-$ 0.593	0.58592	
$\mu$	$-$ 1.55E$-$02	9.89E$-$01	3.57E-04	$-$ 0.016	0.99973	
$\tau$	6.00E-03	1.12E+00	7.08E+00	0.005	0.99589	
generation $\times \mu$	$-$ 3.70E$-$04	3.96E$-$06	6.38E+06	$-$ 93.549	$< $ 2.0E$-$16	***
generation $\times \tau$	$-$ 1.31E$-$04	3.96E$-$06	6.38E+06	$-$ 32.993	$< $ 2.0E$-$16	***
$\mu \times \tau$	3.31E$-$04	7.69E$-$06	6.38E+06	43.089	$< $ 2.0E$-$16	***
generation $\times \mu \times \tau$	7.92E$-$06	1.34E$-$07	6.38E+06	59.030	$< $ 2.0E$-$16	***
**Sensorimotor complexity**						
(Intercept)	1.72E+01	1.62E+00	1.42E$-$01	10.629	0.56900	
generation	1.38E$-$02	4.29E$-$03	3.06E$-$01	3.209	0.48600	
$\mu$	$-$ 1.00E$-$02	2.16E+00	3.31E$-$03	$-$ 0.005	1.00000	
$\tau$	$-$ 1.49E$-$02	2.12E+00	8.10E$-$02	$-$ 0.007	0.99800	
generation $\times \mu$	$-$ 2.75E$-$04	7.63E$-$06	6.38E+06	$-$ 36.018	$< $ 2.0E$-$16	***
generation $\times \tau$	$-$ 3.30E$-$04	7.63E$-$06	6.38E+06	$-$ 43.247	$< $ 2.0E$-$16	***
$\mu \times \tau$	6.55E$-$04	1.48E$-$05	6.38E+06	44.210	$< $ 2.0E$-$16	***
generation $\times \mu \times \tau$	5.67E$-$06	2.58E$-$07	6.38E+06	21.955	$< $ 2.0E$-$16	***

### Morphological complexity

**Fig. 6:**
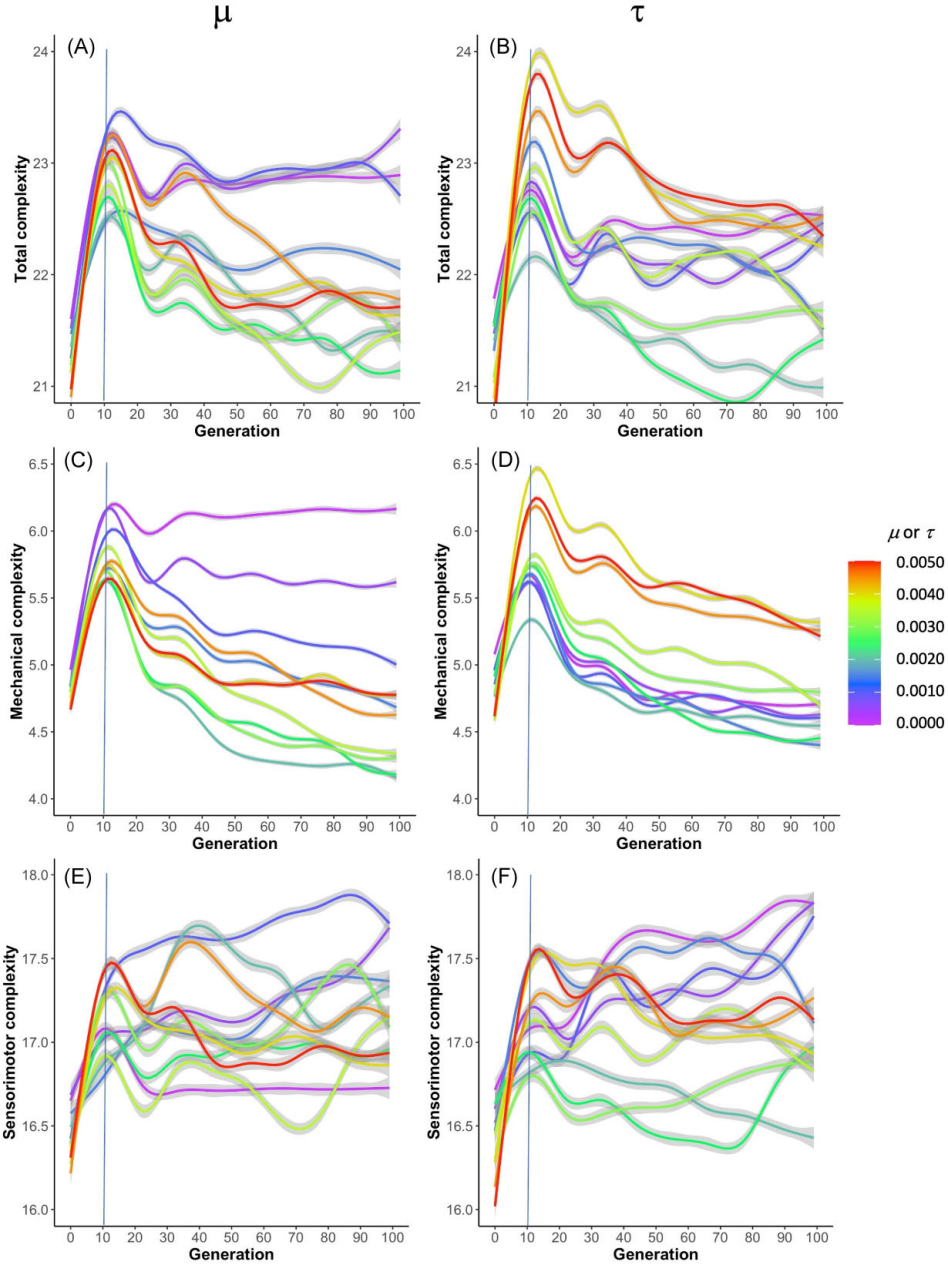
The evolution of morphological complexity differs by type of randomness. Morphological complexity increases rapidly, over the first few generations, when selection is applied to a population of randomly generated individuals. Total complexity (**A**–**B**) is the quantity of all body segments, touch sensors, neurons, and wires. Mechanical complexity (**C**–**D**) is the quantity of body segments. Sensorimotor complexity (**E**–**F**) is the quantity of touch sensors, neurons, and wires. The 95% confidence interval surrounds each line.

As the populations evolve, all three of our metrics for morphological complexity are affected by generational time and the different rates of $\mu$ and $\tau$ (Fig. 6), as indicated by significant three-way interactions (Table 1). For the sake of visual clarity, we illustrate the two-way interactions, which are also significant. With both $\mu$ and $\tau$, every type of morphological complexity rises quickly under selection over the first 10–15 generations, and then it typically reaches a plateau or decreases as evolution continues; decreasing complexity is less common with sensorimotor complexity than with our other complexity metrics. There are differences between effects of $\mu$ and $\tau$, however, regarding the relationship between the magnitude of randomness present and the effects on morphology. For example, low rates of $\mu$ (blue and purple lines) are more associated with high mechanical complexity than low rates of $\tau$ are, whereas high $\tau$ rates (yellow, orange, and red lines) are more associated with high mechanical complexity than high $\mu$ rates are (Fig. 6C and D).

### Variance

**Fig. 7:**
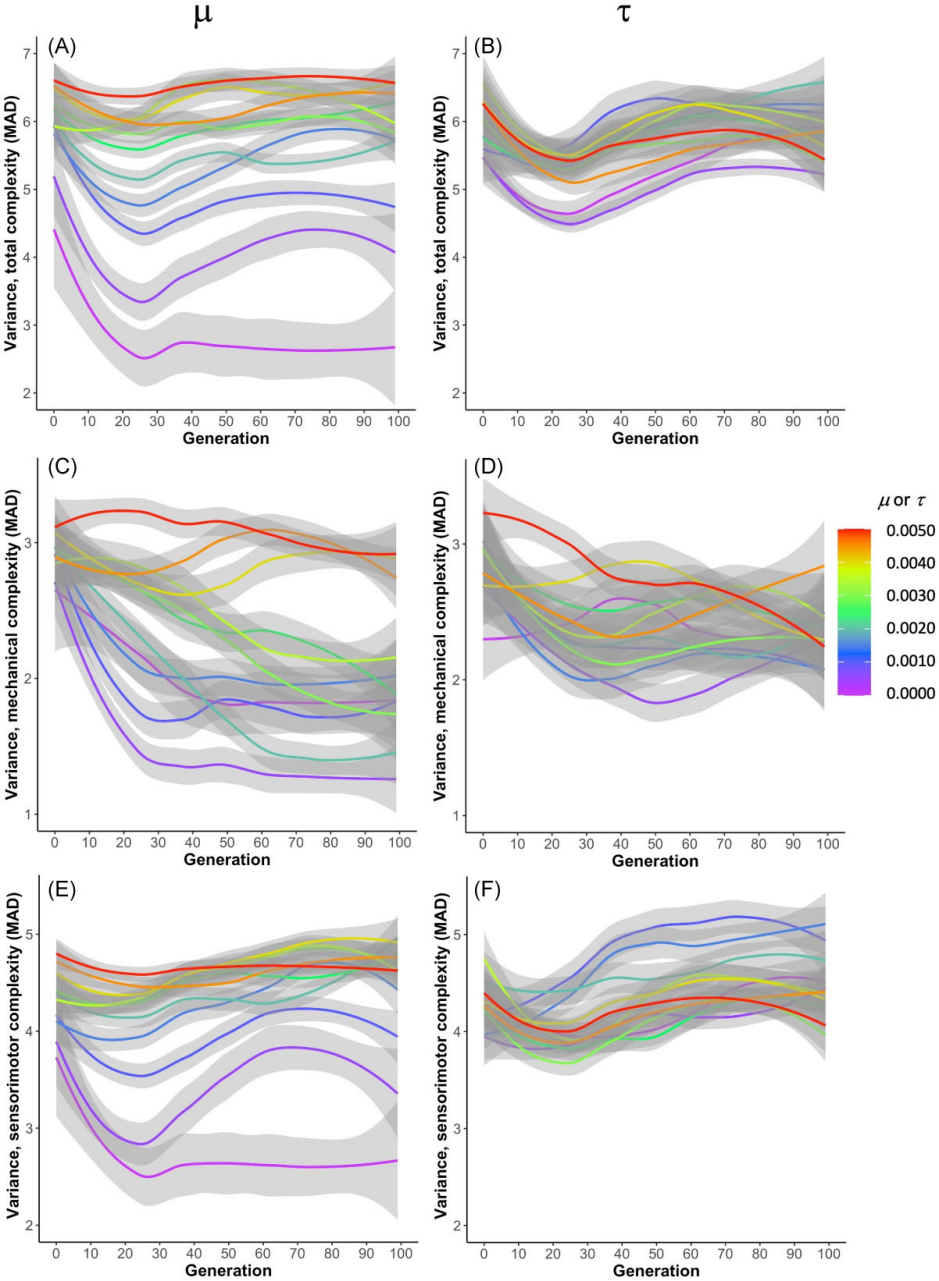
The evolution of phenotypic variance differs by type of randomness. In each population of 60 biorobots, phenotypic variation is measured as the median absolute deviation. The phenotypic variance is less variable with changes in $\tau$ compared to those in $\mu$. Phenotypic variance is on average higher when grouped by $\tau$ than when grouped by $\mu$. **(A**–**B**) Variance in total complexity. **(C**–**D**) Variance in mechanical complexity. **(E**–**F**) Variance in sensorimotor complexity. The 95% confidence interval surrounds each line.

Phenotypic variance, measured as the median absolute deviation of the three morphological complexity measures, evolves in our biorobot populations (Fig. 7). The left column ($\mu$) shows a general trend that in later generations, having undergone more iterations of selection and genomic mutation, higher values of $\mu$ tend to be associated with higher variance in all three complexity measures, though that trend is not strictly monotonic—e.g., for mechanical complexity, intermediate $\mu$ rates (green lines) are not necessarily associated with greater variance than low $\mu$ rates (blue and purple lines). This general trend is not present in the right column, however, which shows the effects of $\tau$. Moreover, the range of variances, across all values of $\tau$, is smaller than the range of observed variances across values of $\mu$.

**Fig. 8:**
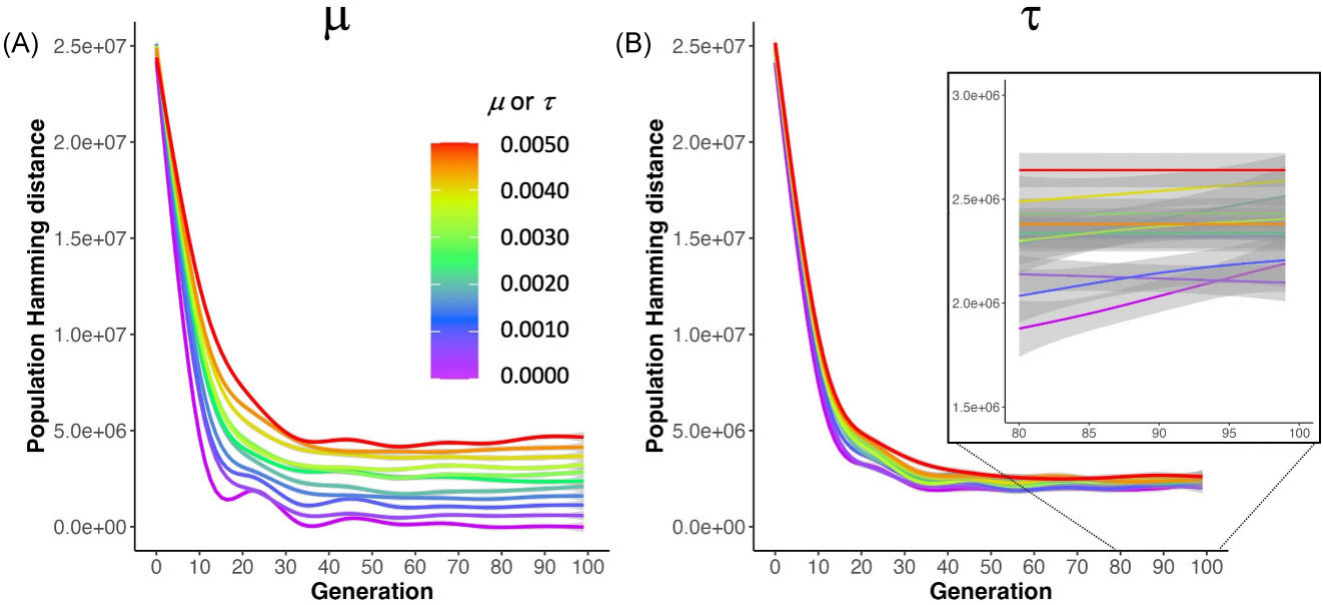
The evolution of genetic variance in populations differs by type of randomness. In each population of 60 biorobots, genetic variation within the population is measured directly as the total Hamming distance among all pairwise combinations of genomes. (**A**) Mutation, as expected, creates genetic variation that is proportional to $\mu$. The 11 levels of mutation $\mu$ are pooled across all levels of $\tau$ for all nine populations ($n = 90$ for each level of $\mu$). (**B**) Transcription error increases genetic variation as the rate of error rises from the lowest to the highest rate of $\tau$. The 11 levels of $\tau$ are pooled across all levels $\mu$ for all nine populations ($n = 90$ for each level of $\tau$). Inset: The lowest values of $\tau$ have lower Hamming distances than the highest values of $\tau$. The 95% confidence interval surrounds each line. A linear mixed-effects model detected significant effects ($p< 0.001$) among all main effects, all two-way interactions, and the three-way interaction among generation, $\mu$, and $\tau$ (Table 1).

Genetic variance of a biorobotic population, measured as the sum of the Hamming distances between the genomes of all individuals, evolves in our biorobotic populations, and does so in different ways depending on the type of randomness examined (Fig. 8) and the significant three-way interaction of generation, $\mu$, and $\tau$ (Table 1). As expected from mutation of the germline, higher rates of $\mu$ correspond straightforwardly to greater genetic variance. The results of randomness in transcription $\tau$, however, are perhaps more interesting and less intuitive. After the initial decrease of variance (ending somewhere around generation 30), $\tau$ values are proportional to genetic variances, even though transcription errors do not directly alter genomes. Overall, however, the magnitude of the effect of $\tau$ on genetic variance is less than that of $\mu$. Finally, the rapid reduction in genetic variance over the first 30 generations is consistent with expectations about the loss of variance in populations that are initially not under selection (generation 0) being subjected to strong directional selection.

### Adaptive landscapes

**Fig. 9:**
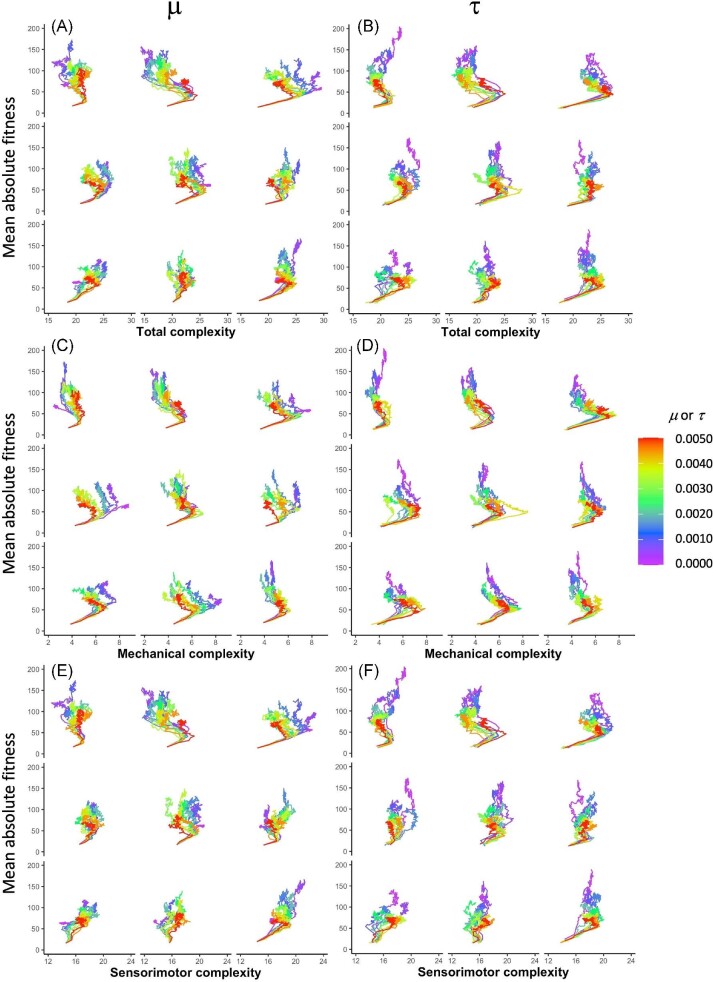
Simple adaptive landscapes of morphological complexity shift with changing types and levels of randomness. For all three types of morphological complexity, the adaptive landscape is the mean fitness of the population plotted against the mean complexity of the population, represented as a path. In each plot, data are clustered by population, with each of the nine populations showing the different evolutionary runs color-coded by either $\mu$ or $\tau$.

**Fig. 10:**
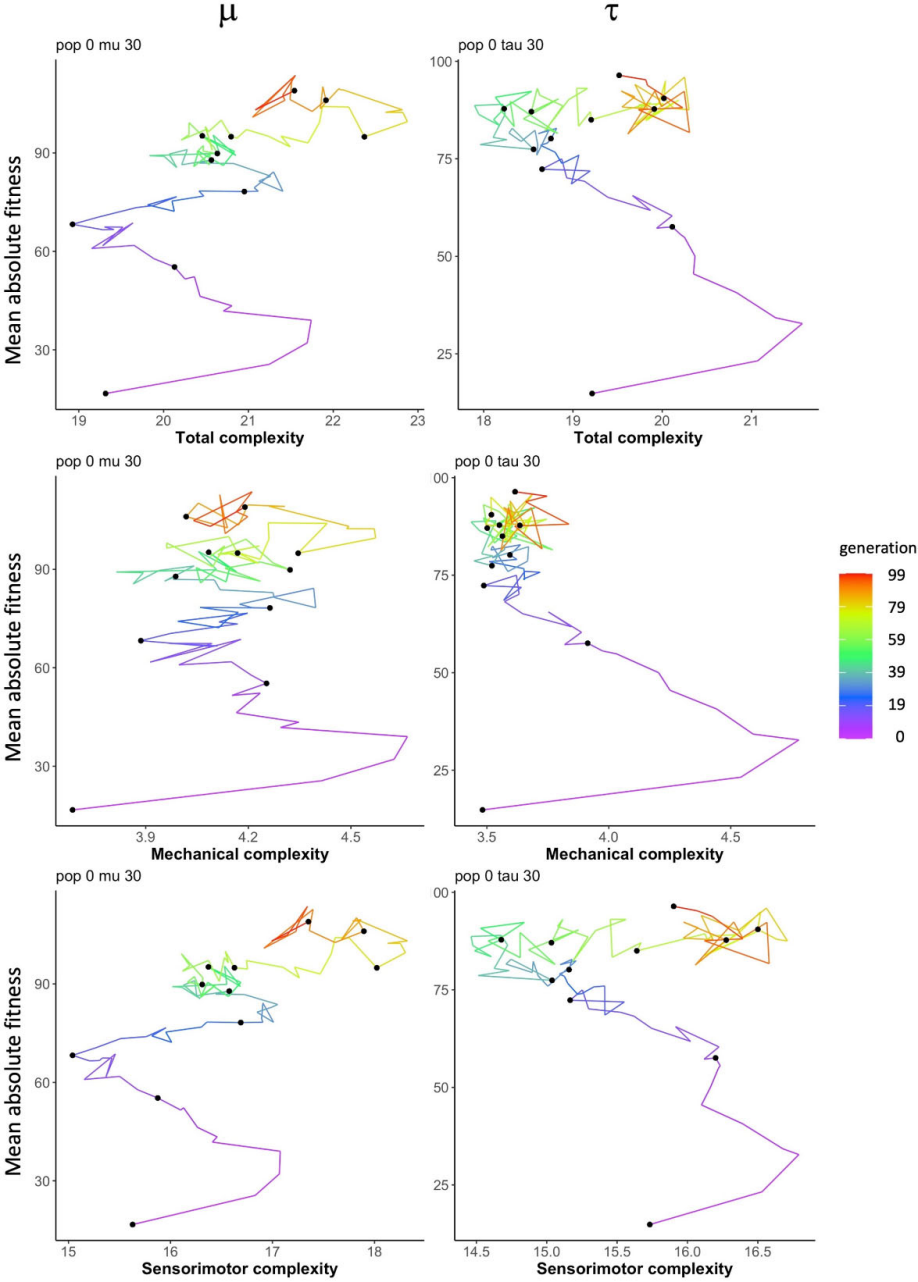
Paths through the adaptive landscape of different complexities: time parameterized. To illustrate the individual behavior of populations in the adaptive landscape of different types of complexity, population 0 is shown with a $\mu$ of 0.0030 (left column) and a $\tau$ of 0.0030 (right column). The generational time is parameterized by color in each path, and points indicate generation from 0 to 99 in increments of 10 generations. Note that the range of the axes differs between plots. Given that mechanical and sensorimotor complexity are non-intersecting subsets of total complexity, the increases in total complexity in the last 40 generations can be seen to result from the underlying increase in sensorimotor complexity. The late-stage increase in sensorimotor complexity is not correlated with an increase in fitness.

The relationships between a population’s mean absolute fitness and its mean morphological complexity are complicated, even in simple adaptive landscapes (Fig. 9). In each panel, the data are clustered by each of the 9 populations, with all 11 of that population’s evolutionary runs color-coded by either $\mu$ (left column) or $\tau$ (right column). We call each run a *path* in this context, and each path is temporal, with time parameterized along it (Fig. 10), such that no matter the population, type of complexity, or rate of $\mu$ or $\tau$, each path starts at low complexity and low fitness.

Across all conditions, there is typically, but not always (as noted below), an initial burst of increasing morphological complexity and fitness in response to selection (Fig. 9). This is often, but not always, followed by a decrease in complexity and a continued rise in fitness. Note that for diagrams grouped by $\mu$ value, every population starts at the same point, reflecting the same morphology and fitness—genomic mutation has not yet occurred for that initial population, and by pooling over all $\tau$ values, all effects of randomness in development are initially identical for each population—but those measures diverge as populations evolve. For diagrams grouped by $\tau$ value, however, starting points differ, reflecting different rates of randomness in the developmental process that resulted in the initial generation, and unaffected by $\mu$ because genomic mutation has not yet occurred.

Paths through the adaptive landscape show the conditions under which extremal values of fitness and morphological complexity occurred and how they differ by type of randomness and type of complexity (Fig. 9). When considering occurrences of maximal complexity for both mechanical and sensorimotor morphologies, maximal sensorimotor complexity tends to be associated with higher fitness values than maximal mechanical complexity does. (Values of sensorimotor complexities are substantially greater than those of mechanical complexity, due to the definitions of our complexity metrics.) In addition, when considering data grouped by $\tau$ value, the majority of populations attained maximal mechanical complexity at intermediate or high $\tau$ values (colors between green and red), whereas the majority of populations attained maximal sensorimotor complexity at low $\tau$ values (blue or purple). When considering data grouped by $\mu$ value, however, that pattern does not hold—for example, for mechanical complexity, the majority of populations attained maximal complexity at *low*$\mu$ values. These generalizations apply to the majority of populations, but not to all: Whether data are grouped by $\mu$ or $\tau$, for both mechanical and sensorimotor complexity, there were multiple populations that did not conform with those generalizations, including some in which low and high randomness values both came close to the maximal complexity attained by the population.

When considering occurrences of maximal fitness, rather than complexity, across all populations, complexity metrics, and conditions of randomness, maximal fitness does not tend to occur at an extreme of complexity (i.e., either maximal or minimal complexity). There are exceptions—e.g., the top-left population in Fig. 9, showing sensorimotor complexity grouped by $\tau$—but as a tendency, maximal fitness occurs away from extremal complexity. Moreover, when comparing $\mu$-grouped data to $\tau$-grouped data, the lowest $\tau$ values (purple) are uniformly associated with maximal fitness, whereas for $\mu$, low to intermediate values (blues and greens) are often associated with maximal fitness. High rates of randomness are never associated with maximal fitness in our populations.

### Selection gradients

After the initial burst of increasing morphological complexity and fitness (Fig. 9), the changing correlations between morphological complexity and fitness over generational time are not readily predictable, so we performed selection gradient analysis for additional insight. As a general technique, selection gradient analysis indicates the extent to which traits are targeted by selection over generational time (Arnold 1983); here, we applied it to each population, with respect to morphological traits that were defined to correspond to our mechanical and sensorimotor complexity metrics—the model from which selection gradients were generated was ${fitness} = A + B_1 \cdot {{mechanical\_complexity}} + B_2 \cdot {sensorimotor\_complexity}$, where *B* are the unstandardized coefficients in a multivariate least-squares regression. Because total morphological complexity is not independent of the other complexity measures, it was not included.

**Fig. 11:**
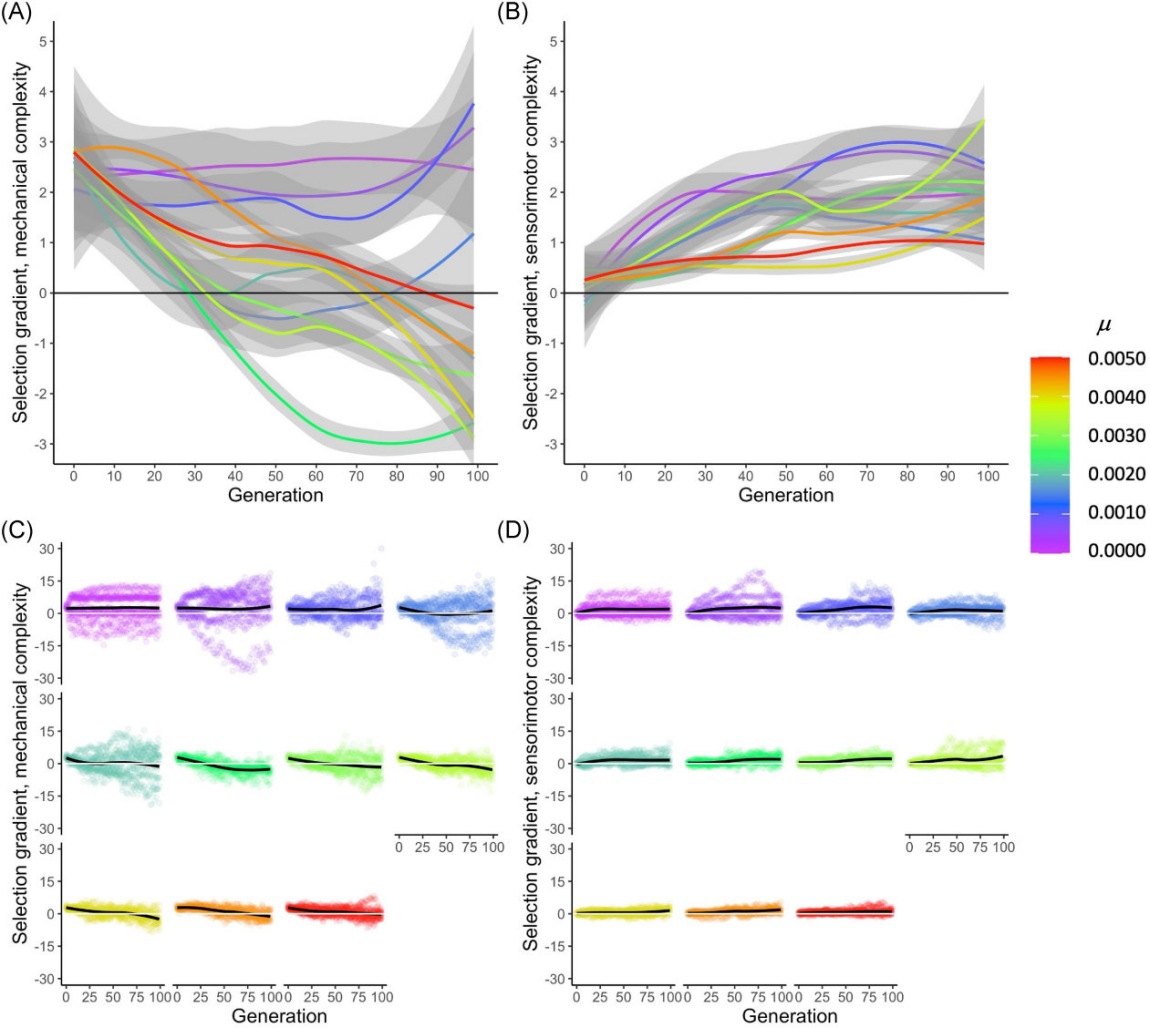
Selection gradients for mechanical and sensorimotor complexity change as a function of $\mu$ and time. **(A)** Regression lines for mechanical complexity are initially positive across all levels of $\mu$, but become strongly negative at intermediate levels of $\mu$ after generation 30. **(B)** Regression lines for sensorimotor complexity remain positive across all levels of $\mu$ and over all generations. Data in **(A**–**B)** are pooled over all 11 levels of $\tau$. **(C)** For mechanical complexity, decreases in the selection gradients over generational time are clearly seen at intermediate levels of $\mu$. Note that the points show a wide range of positive and negative gradients, and that range is smallest at the highest $\mu$. **(D)** For sensorimotor complexity, increases in the selection gradients over time are small and consistent. Ranges, as shown by points, decrease with increasing $\mu$.

**Fig. 12:**
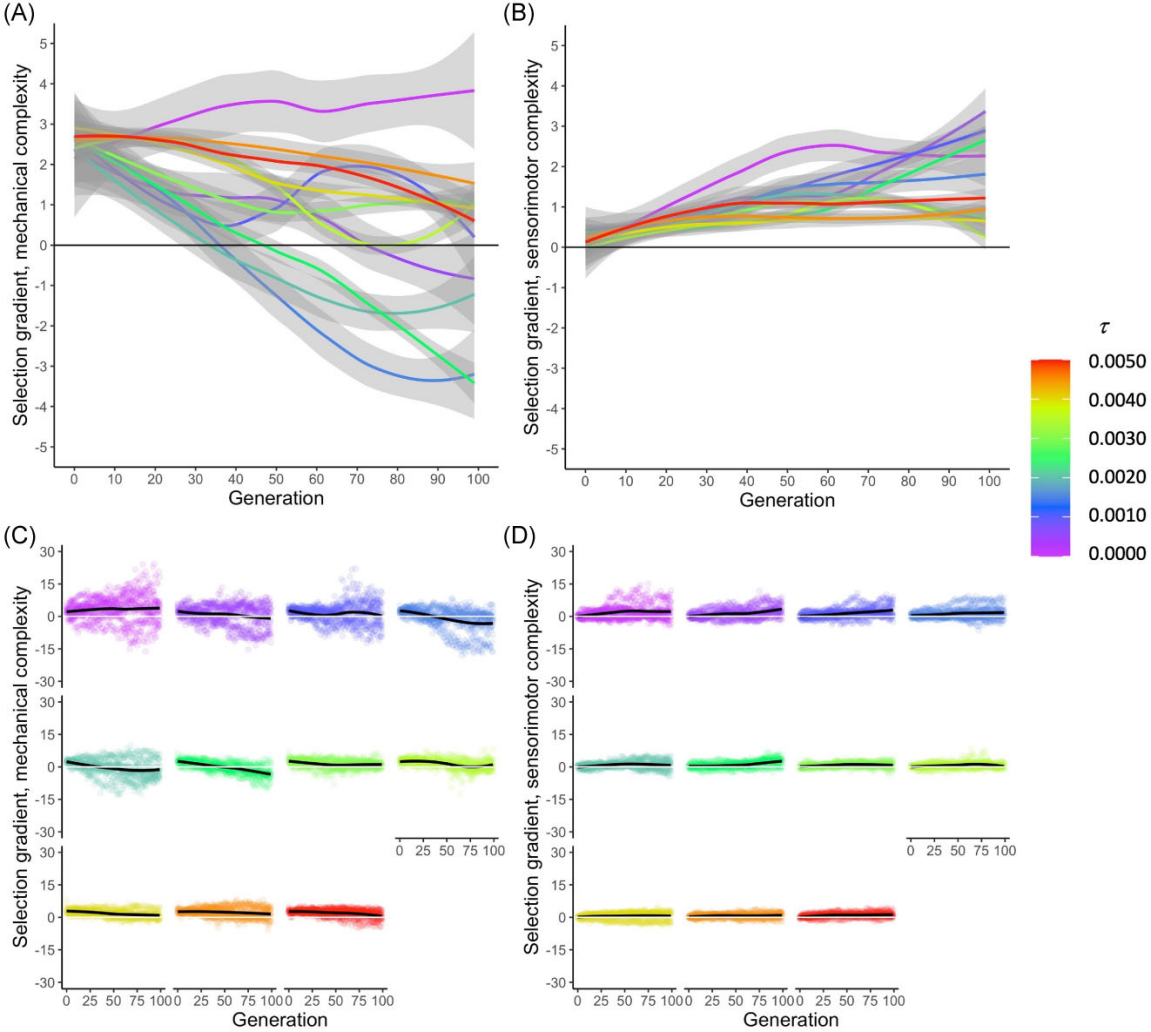
Selection gradients for mechanical and sensorimotor complexity change as a function of $\tau$ and time. **(A)** Regression lines for mechanical complexity are initially positive across all levels of $\tau$, but then become strongly negative at intermediate levels of $\tau$ after generation 30. **(B)** Regression lines for sensorimotor complexity remain positive across all levels of $\tau$ and over all generations. Data in **(A**–**B)** are pooled over all 11 levels of $\mu$. The 95% confidence interval surrounds each line. **(C)** For mechanical complexity, decreases in the selection gradients over generational time are seen at intermediate levels of $\tau$. Note that the points show a wide range of positive and negative gradients, and that range is smallest at the highest levels of $\tau$. **(D)** For sensorimotor complexity, increases in the selection gradients over time are small and consistent. Ranges, as shown by points, decrease with increasing $\tau$.

Whether selection gradients are grouped by $\mu$ or $\tau$ value, differences in how selection is targeting mechanical and sensorimotor complexity are apparent (Figs. 11 and 12). For instance, selection targets increasing mechanical complexity in early generations for all $\mu$ values, but by generation 30, selection gradients are negative for intermediate $\mu$ values (green lines), and by generation 100, selection gradients are substantially negative for both intermediate and high $\mu$ values (Fig. 11A). Different dynamics, however, occur with sensorimotor complexity (Fig. 11B). Sensorimotor complexity starts out not being strongly targeted, but selection gradients have typically increased by generation 100; moreover, in contrast to mechanical complexity, none of the regression lines for sensorimotor complexity show negative values in later generations.

More details emerge when all of the data points are plotted (Figs. 11C, D): For both mechanical and sensorimotor complexities, for *every*$\mu$ value, some selection gradient values are indeed negative. Nonetheless, these data still support the observed general trend: For intermediate and high $\mu$ values, mechanical complexity becomes less positively targeted by selection over the generations studied, to the point where selection tends to target *against* increasing numbers of segments in later generations; in contrast, for sensorimotor complexity, targeting is generally more positive at generation 100 than at generation 0.

We see similar patterns in selection gradients grouped by $\tau$, with some regression lines with negative values for mechanical complexity in later generations (Fig. 12A), but none for sensorimotor complexity (although one comes close; Fig. 12B). When individual points are examined (Figs. 12C, D), we see some negative selection gradients in all conditions, along with the trends indicated by regression lines.

## Discussion

The *ECE* modeling framework enables investigations of interactions among genetics, development, behavior, and selection (Hawthorne-Madell 2021); effects of developmental constraints and selection on the evolution of morphology (Aaron et al. 2022); and as demonstrated in this paper, effects of different types of randomness—germline mutation and SDV (Vogt 2015)—on the evolution of morphological complexity. All three of these studies use the same ECE model and its set of experimental results, a database of over 6 million individual biorobots that developed, behaved autonomously, and, following selection, reproduced as members of 9 replicate genome populations evolving over 100 generations of selection.

Our experimental results, as shown by this new analysis, failed to refute the predictions associated with our two primary hypotheses:

- ***H_1_***: Randomness in the gene transcription process alters the direct impact of selection on populations.
- ***P_1_***: Variations in the rate of randomness in the gene transcription process will vary the dynamics of selection (Fig. 12).
- ***H_2_***: Selection on locomotor performance targets morphological complexity.
- ***P_2_***: The morphological complexity of populations will evolve adaptively, as detected by the presence of non-zero selection gradients (Figs. 11 and 12).

The support here for the first hypothesis adds SDV to the list of types of randomness that drive evolution, along with well-known sources such as mutation, recombination, genetic drift, associative mating (for review, see Bonner 2013; Lynch 2007). But the relative impacts of the various modalities of randomness remain to be fully explored. Random transcription errors propagate through development to create phenotypes that can imperfectly represent the codons in a given individual’s genome. The resulting effect on selection (prediction ***P_1_***) is not limited to transcription, according to the *MGH* in its broad sense (Hawthorne-Madell 2021): Any process in the genome-to-phenome mapping that is not completely deterministic could alter selection of individuals and the evolution of populations. Motivated by the observation that development has both serial and parallel processes operating concurrently, ECE modeling enables testing of the MGH in the explicit context of an interaction network, with multiple processes operating with various rates of random error generation. Relative impacts of different parts of the network could be tested and then, once understood, compared with other modalities of randomness outside of the G-to-P map.

The support here for the second hypothesis helps advance our understanding of the adaptive value of morphological complexity. Using the quantity of similar elements in segmented organisms as a metric of complexity (“horizontal complexity,” in the sense of McShea 2021), our model provides evidence that selection on behavior—here, locomotor performance—can directly target complexity (prediction ***P_2_***). How, when, and whether complexity is adaptive is an intriguing set of questions, with answers likely dependent on the specifics of the evolving system and the ways in which complexity is measured. For example, complexity is often defined as the number of different *types* of elements in a system, (“vertical complexity,” in the sense of McShea 2021), with more diversity of types increasing complexity. To test the adaptive value of this type of complexity, ECE models could be created that, unlike the one in this study, allow different types of parts to evolve.

For this study, ECE modeling enabled us to conduct 121 separate experiments—121 different combinations of mutation rate $\mu$ and transcription error rate $\tau$ in a factorial design—on the same replicate genome populations. This control over independent variables provides the scope and granularity to simultaneously investigate broad trends and subtle differences. But a model is merely a representation of a target system. With biological organisms, the only system that comes close to the scope and granularity of the computational modeling approach are long-term evolutionary experiments on *Escherichia coli* bacteria (Lenski and Travisano 1994 and Lenski 1999). Thus, we first discuss caveats and trade-offs of computational modeling in general, and ECE in particular, before discussing the details of our experimental results.

### Caveats and trade-offs

Every model and every experiment involves decisions and trade-offs: You can’t do it all, so what do you choose to do? The power of a model, formal or physical, comes from its ability to explicitly represent parameters of interest in the target system and vary them systematically (Webb 2001), providing control over independent variables for the type of comprehensive exploratory research characterized as *integrative experimental design* (Almaatouq et al. 2022). ECE is conceptually dedicated to conducting experiments on populations of behaviorally autonomous, self-propelled organisms, biorobotic models with realistic morphology, and reconfigurable bodies. With its focus on individuals from which, collectively, higher-order phenomena emerge, ECE models are agent-based, inspired by the experimental evolution of physical biorobots (for review, see Long 2012) and digital organisms (Lenski 1999).

In this paper, our ECE model enables precise control over rates of germline mutation $\mu$ and transcription error $\tau$, but like all computational models, it has limitations that entail trade-offs (Table 2). All of these trade-offs are apparent in the experiments presented in this paper, with perhaps the most important being that the organisms, and the biological phenomena associated with them, are simulated (Webb 2001). Simulated organisms and simulated environments introduce a “reality gap” (see Jakobi et al. 1995), raising questions of validity concerning what we learn about the modeled organisms and what we intend to learn about the actual life forms. Thus, any biorobotic modeler—whether working with simulated organisms *in silico* or those that are physically realized (Long 2012)—makes decisions about which parts of the target biological system are to be represented in the model, how those parts will be simplified to enable mathematical rendering or mechanical construction, and how to give the simulated organism the autonomous behavior that defines the original scientific biorobotic approach (Webb 2001).

**Table 2:** Trade-offs with the ECE Modeling Methodology

**Pros:**
1. Explicit, precise control over experimental variables: Both independent and dependent variables are predefined and algorithmically manipulated and measured.
2. Explicit, precise control over other parts of the model: Parameters and functions can be fully experimentally controlled, including parameters such as population size, number of generations, number of genes, and functions such as mutation, reproduction, development, and selection.
3. Complete populations: Every individual is known and studied.
4. Explicit starting conditions: Historical effects are known and determined.
5. Omniscience: All aspects of the system can be known as it unfolds over the simulated time.
**Cons:**
1. Organisms and other biological processes are simulated: Validity is limited since computational representation is not the real biological system.
2. Simplification: Models of agents, biological processes, and their environments represent only a subset of all possible aspects of the biological system.
3. Abstraction: Representations at one structural or process level may only implicitly represent mechanisms at other levels of the target biological system.
4. Starting conditions: Where to begin may be arbitrary with respect to the complete evolutionary history of the biological system.
5. Unintended consequences: Although it may be infeasible to critically consider every possible consequence of design decisions, the modeler bears responsibility for unintended consequences that can alter the model’s validity.

As one source of unintended and unexplored consequences for our ECE model in this paper, consider the genetic code of our biorobots (Fig. 2). It is not biologically inspired in at least these respects: (1) a sphere (segment) transcript, but not other parts, is redundantly coded (000, 001); (2) feature codons, which determine anatomically where and whether the parts are connected to each other, occupy the majority of the available codon space; and (3) the neighborhood structure of the codons biases the probability that any single point mutation will cause a particular change in a codon and the transcript it produces. For example, a single point mutation can change a sensor (100) into a sphere (000), joint (200), joint feature (300), sphere sensor x feature (110), sphere sensor z feature (120), wire connections feature (130), or sensor output feature (101, 102, 103). But genomic changes are not all equiprobable from a given codon, and we did not intentionally design that aspect of the model. If one were interested in the consequences of codon neighbor structure, it could be explored experimentally in a follow-up study within the ECE framework.

### Randomness of different types as causal processes

Commonly, evolutionary biologists model randomness as a causal mechanism in the form of either genetic drift or germline mutation. Both can be abstracted mathematically in concert with selection to produce the fundamental models of population genetics (Hartl 2020). From population genetics, we know the role played by random mutation in quantitative traits: It is a source of additive genetic variance, a feature of the population that allows a phenotypic trait to be both heritable and responsive to selection. What’s less often appreciated is that heritable phenotypic variance, essential for the operation of natural selection, can also be generated by non-mutational mechanisms (Payne and Wagner 2019). Using this ECE model, we previously demonstrated (Hawthorne-Madell 2021) that by adding randomness to the mapping between genotype and phenotype in development, an SDV mechanism can, over generational time, protect and even restore genetic variance, which is foundational for heritable phenotypic variance.

By comparing the rates and effects of mutation $\mu$ and transcription error $\tau$ in a factorial experimental design, we gain insight into the parallel workings of, and interactions between, these different evolutionary drivers. As we have shown in the analysis of our experiments, these two different types of randomness act in similar and different ways on the evolution of morphological complexity over generational time. Statistical modeling—while not required for inference in situations like these where all individuals are known and measured—permits a concise summary of the interactions of the experimental factors (Table 1). Different rates of $\mu$ and $\tau$ systematically alter the evolutionary patterns of total and mechanical complexity and, to a lesser degree, sensorimotor complexity (Fig. 6).

Although $\mu$ and $\tau$ both alter the adaptive dynamics of the populations, they do so differently. Focusing on mechanical complexity, note that the highest levels of $\mu$ produce intermediate levels of complexity, while the highest levels of $\tau$ produce some of the highest levels of complexity (Fig. 6C, D). Moreover, the lowest levels of $\mu$ produce the highest levels of complexity, while the lowest levels of $\tau$ produce the lowest levels of mechanical complexity. These evolutionary differences are explained, in part, by the effect of the randomness on the populations’ phenotypic variance. Genetic mutation must be at high levels in order to sustain phenotypic variance and a response to selection over the 100 generations of selection (Fig. 7C). This may explain why mechanical complexity does not change after generation 50 at the lowest levels of $\mu$ (Fig. 6C): That stasis results from a loss of phenotypic variance (Fig. 7C), even though genetic variance is steady and non-zero (Fig. 8A). Without much phenotypic variance, selection on behavior cannot act strongly on that trait. Given that selection gradient analysis shows that selection on mechanical complexity remains strong and positive across all generations at the lowest levels of $\mu$ (Fig. 11A), we know that the populations at low levels of $\mu$ have lost the ability to respond to selection.

In contrast to $\mu$, variations in $\tau$ alter only slightly the phenotypic variance in mechanical complexity over 100 generations of selection (Fig. 7 D), allowing populations across the levels of $\tau$ to steadily evolve reduced mechanical complexity (Fig. 6D). Although our data show phenotypic variance is not strongly driven by $\tau$, selection gradient analysis shows that $\tau$ varies the selection gradients on mechanical complexity (Fig. 12A), with four of the lowest rates changing from positive to negative; the remaining rates, along with $\tau = 0$, are associated with positive selection gradients over the 100 generations. We see a similar but not identical pattern in the selection gradients with respect to $\mu$ (Fig. 11A), which points to the differences in the effects of $\tau$ and $\mu$ on phenotypic variance (Fig. 7) as determining factors in their differences on the evolution of morphological complexity (Fig. 6).

In comparison to mechanical complexity, sensorimotor complexity varies over a smaller range, about one element compared to nearly two elements (compare Fig. 6E and C), in spite of the fact that the magnitude of sensorimotor complexity is greater than that of mechanical complexity. Moreover, the variations over time for sensorimotor complexity are more variable for any given level of $\mu$ or $\tau$ and less consistent across levels of $\mu$ or $\tau$. Note also the differences in how selection acts upon them: The selection gradients are uniformly positive, after generation 10, for sensorimotor complexity under variations in $\mu$ (Fig. 11) or $\tau$ (Fig. 12). Thus, in terms of evolutionary dynamics, these are two different types of complexity. Overall, germline mutation and transcription error work in different ways to alter the rate and direction of a population’s evolution.

### Genetic history and evolution of populations

At the level of individual populations in a simple adaptive landscape, we saw the three possible patterns for fitness and complexity over generational time in our data (Fig. 9):

1. fitness and complexity remain positively correlated,
2. they become negatively correlated, or
3. they become decoupled.

These differences are correlated with the population, not with the type or rate of randomness, suggesting that starting genomic conditions predispose a given long-term response to selection.

No matter the rate of $\mu$ and $\tau$, all of the populations rapidly increase total, mechanical, and sensorimotor complexity in the first 10 generations (Fig. 9). We were surprised that this first burst of evolution would be an invariant pattern, largely independent of the levels of $\mu$ and $\tau$, since both the genetic and phenotypic variance that $\mu$ and $\tau$ create, and which vary by design in the experiment, are essential ingredients for adaptation in standard evolutionary theory (for review, see Lynch 2007). But surprises have also been seen in ecological systems: When rapid bursts follow speciation events, they are seen as paradoxical accelerations with respect to the deceleration predicted by many models (Martin and Richards 2019). Alternatively, this can be seen as an initial convergence or consolidation from a completely random system to one that is structured by its interaction with a common environment and a common selection pressure.

For our biorobotic populations, we have the information and precise experimental control to investigate and understand what is occurring. Most importantly, each of the nine biorobotic populations starts with 60 genomes that are random with respect to the selection environment; this situation is roughly analogous to a small founding population translocated into a new ecological context. But the biorobotic populations do not suffer from reduced genetic variance relative to the sub-sampled parental populations, or from the resulting initial slow response to selection known as the *founder effect* (Roman and Darling 2007). Instead, the genetic variance in each starting population, by virtue of being created randomly, represents not a sub-sampling of a parental population but rather a sampling distributed across all possible genomes. The rapid burst of evolution that follows selection quickly winnows, via truncation, those 60 genetically disparate asexual lineages down to the 30 that reproduce in that first generation. Thus, in just a few generations, selection shifts from acting on the variance provided by different founding lineages of asexual biorobots to the genetic variance provided by mutation and the masquerading genome effect (Hawthorne-Madell 2021) of transcription accumulating variation within just a few selected lineages.

A shifting intensity and direction of selection gradients over time (Figs. 11 and 12) could suggest that the system’s adaptive landscape is shifting over time ([Bibr bib20][Bibr bib20]), or that other causal mechanisms are in parallel play. To investigate $\mu$ and $\tau$ in this context, we examined separate one-dimensional adaptive landscapes for total, mechanical, and sensorimotor complexity (Fig. 9). For most populations and levels of randomness, the pathways through the landscape show similar beginnings, from low complexity and fitness to higher complexity and fitness. But a variety of patterns may occur within a given population over time (Fig. 10), or under different rates of randomness.

Even with these divergences, we can see similarities in evolutionary behavior within individual populations. For example, population 3 has, for all combinations of complexity and types of randomness (upper right-hand corner of panels in Fig. 9), the greatest initial increase in fitness among the populations. After the initial strong positive correlation between complexity and fitness, this correlation reverses and complexity declines as fitness rises. Contrast the evolutionary behavior of population 3 with that of population 1 (top left of each panel in Fig. 9), which achieves the highest mean fitness of the populations by decoupling complexity and fitness at low to intermediate levels of $\mu$ and $\tau$.

Since each population has a distinctive evolutionary behavior, independent of experimental condition, it is tempting to think of each population as having a “personality”—the effects of the population’s particular genetic history. For each population, its genetic history begins with a single set of 60 randomly generated genomes, the same starting point in each of the 121 conditions. The differences among its 121 evolutionary trajectories start with transcription errors that, before selection acts or mutation operates, immediately create masquerading genomes (Hawthorne-Madell 2021), with some randomly altered phenotypes that vary from the deterministic mapping of their genotypes. After selection and through reproduction, mutation and differential reproduction create genetic differences in the population’s next generation. In this manner, within and between generations, $\tau$ and $\mu$ drive different paths that the same population takes under steady directional selection.

### Evolution of morphological complexity

Constant directional selection for enhanced locomotion involves different solutions, consistent with the diversity of body forms evolved (Fig. 4). In a previous study on physical biorobotic models (Roberts 2014), selection, as measured by selection gradients (Aaron et al. 2022; Arnold 1992), switched phenotypic targets from one trait to another. Similar dynamics are occurring here, but the situation in this paper is far more complicated, with larger populations, 100 generations, and 121 experimental conditions.

During the early stages of evolution, generations 0–30, selection gradients on mechanical and sensorimotor complexity are positive, no matter the level of $\mu$ (Fig. 11) or $\tau$ (Fig. 12). For later generations, that pattern of positive selection gradients continues only for sensorimotor complexity (Figs. 11B and 12B). For mechanical complexity, the selection gradients switch dramatically for intermediate levels of $\mu$ (Fig. 11A), with some but not all low and intermediate levels of $\tau$ becoming strongly negative (Fig. 12A). For intermediate levels of $\mu$, the change in selection gradients on mechanical complexity over generation has a consistent negative slope (Fig. 11C); this pattern is present but more variable at intermediate levels of $\tau$ (Fig. 12C).

Given that selection acts differently on mechanical and sensorimotor complexity, these two traits may evolve independently or in concert. Thus, populations may evolve to have all combinations of mechanical and sensorimotor complexity, with the probability for a particular outcome predicted by the underlying rates of $\mu$ and $\tau$ (Fig. 6). When viewed across the experimental conditions of $\mu$ and $\tau$ (Fig. 10), the increasing divergence among lineages (biorobotic populations here) over time is consistent with expectations from the *zero force evolutionary law (ZFEL)*: non-adaptive, random differences will accumulate in lineages over time even in the presence of selection (McShea et al. 2019). Please note that we did not directly test for ZFEL in our experiments. Also consistent with ZFEL, we have previously shown in these biorobots that the irreversibility of their development imposes a tendency to bias random walks in segment-branch morphospace toward increasing mechanical complexity over generational time (Aaron et al. 2022).

We parsed total complexity into mechanical and sensorimotor components because of their different functional roles; note that counting the number of parts, as we’ve done here, is a simple metric of “horizontal” complexity, which focuses on one level of an organism’s hierarchical design (McShea 2021). Because these component systems consist of parts made from distinct genes (Fig. 2), they can evolve independently. They do so under some conditions: Their evolutionary trajectories diverge at intermediate values of $\mu$ or $\tau$, when the selection gradients are negative on mechanical complexity (Figs. 11 and 12). This result suggests how different quantitative traits in general, and the different complexities in particular, may diverge within a population under steady directional selection acting on locomotor performance.

Thus, the ECE approach has shown the ability to answer McShea’s call (McShea 2021) for an understanding of complexity that allows us (1) to measure it in these simplified biorobots and (2) to measure changes in that complexity over evolutionary time. With conceptual roots in “artificial selection” (Darwin 1964), ECE is aligned methodologically with *experimental evolution*, the long-term selection of individuals under reproducible conditions in populations of bacteria (Rebolleda-Gómez and Travisano 2019; Travisano et al. 1995), digital organisms (Lenski 1999), and plants and animals (for review, see Garland and Rose 2009). By augmenting experimental evolution with computational models for studying embodied individuals and populations—with complete access to their genetics, development, behavior, and evolutionary dynamics (Fig. 1)—ECE could be a welcome methodological addition for investigators of evolution.

## Data Availability

The ECE code and the experimental data are available at https://github.com/josh-hm/EvoDevo-Modeling.
